# SILAM for quantitative proteomics of liver Akt1/PKBα after burn injury

**DOI:** 10.3892/ijmm.2011.861

**Published:** 2011-12-14

**Authors:** X.-M. LU, R.G. TOMPKINS, A.J. FISCHMAN

**Affiliations:** 1 Massachusetts General Hospital, Boston, MA, USA; 2 Harvard Medical School, Boston, MA, USA; 3 Shriners Hospitals for Children, Boston, MA, USA

**Keywords:** Akt1/PKBα, SILAM, MS/MS, burn injury

## Abstract

Akt1/protein kinase Bα (Akt1/PKBα) is a downstream mediator of the insulin signaling system. In this study we explored mechanism(s) for its role in burn injury. Akt1/PKBα in liver extracts from mice with burn injury fed with (^2^H_7_)-L-Leu was immunoprecipitated and isolated with SDS-PAGE. Two tryptic peptides, one in the kinase loop and a control peptide just outside of the loop were sequenced via nano-LC interfaced with quadruple time-of-flight tandem mass spectrometry (Q-TOF tandem MS). Their relative isotopologue abundances were determined by stable isotope labeling by amino acids in mammalians (SILAM). Relative quantifications based on paired heavy/light peptides were obtained in 3 steps. The first step included homogenization of mixtures of equal amounts of tissue from burned and sham-treated animals (i.e., isotope dilution) and acquisition of uncorrected data based on parent monoisotopic MS ion ratios. The second step included determination of isotopic enrichment of the kinase from burned mice on Day 7 and the third step enrichment correction of partially labeled heavy and light monoisotopic MS ion ratios for relative quantification of bioactivity (loop peptide) and expression level (control peptide). Protein synthesis and enrichment after injury were found to be dependent on tissue and turnover of individual proteins. Three heavy and light monoisotopic ion ratios for albumin peptides from burned mice indicated ~55% enrichment and ~16.7-fold downregulation. In contract, serum amyloid P had ~66% enrichment and was significantly upregulated. Akt1/PKBα had ~56% enrichment and kinase level in response to the burn injury was upregulated compared with the control peptide. However, kinase bioactivity, represented by the Cys^296^ peptide, was significantly reduced. Overall, we demonstrated that i) quantitative proteomics can be performed without completely labeled mice; ii) measurement of enrichment of acyl-tRNAs is unnecessary and iii) Cys^296^ plays an important role in kinase activity after burn injury.

## Introduction

Stable isotope labeling by amino acids in cell culture (SILAC) provides relative quantification of *in vitro* protein synthesis and functional proteomics under conditions that mimic disease states (1–5). Typically, two cell populations are cultured for six doublings times; control cell medium contains the natural amino acid (e.g., ^12^C_6_-Lys and/or ^12^C_6_-Arg, 99% natural abundance), and the second medium contains the same levels of amino acids with heavy isotopes (e.g., ^13^C_6_-Lys and/or ^13^C_6_-Arg, 98% abundance) and disease related stimuli. The two cell populations are mixed with equal amounts of total protein and digested peptides are accurately measured by mass spectrometry with a mass difference of 6 Da for singly charged parent ions. Six doubling times allows isotopic enrichment of the precursor acyl-tRNA pool to reach ~98% in cancer cell lines. Labeling above 95% is generally required for comparative and quantitative proteomics by MS. Thus, the relative abundance of any paired peptide’s monoisotopic MS ion chromatogram with SILAC can be used to measure protein synthesis in comparison with the controls under *in vitro* conditions. The SILAC approach has been used with *C. elegans* fed with >98% ^15^N-labeled *E. coli* (6), skeletal muscle from chickens fed with a synthetic diet containing 50% of (^2^H_8_)-Val (7,8), partially labeled rat diet with >99% ape ^15^N algal cells for 44 days (9) and F1–F4 offspring of mice fed with a Lys-free diet containing 1% of L-^13^C_6_-Lys (10). Complete labeling has been reported to be achieved by the F2 generation. Metabolic labeling with ^15^N can be performed efficiently and economically, however, data interpretation can be challenging since the monoisotopic peak can shift, due to the distribution of positional isotopomers as a function of labeling time (11,12). Global labeling with ^15^N has been used as a tool for characterization of enrichment under partially labeling conditions (13–15). Stable isotope-labeled amino acids, such as L-^13^C_6_-Lys and L-^13^C_6_-Arg, provide ideal residues at C-terminal labeling positions for trypsin digestion. Protein synthesis depends on 2 factors: acyl-tRNA levels and protein turnover rate. These factors are tissue, cell type, time and treatment dependent. To minimize individual variability, full incorporation of L-^13^C_6_-Lys can be achieved in mice; however, it is very costly.

Analysis of the hypermetabolic/inflammatory response under acute phase conditions is very challenging for several reasons: i) significant changes in protein expression are associated with high levels of reactive oxygen species (ROS) and post-translational protein modifications (PTM); i.e. not only protein levels but also biological activities have to be quantified; ii) individual protein enrichments and incorporated isotope distributions may vary with the partially isotope enriched precursor t-RNA pool (>50%) and corresponding protein turnover rates during the acute phase response.

The metabolic alterations produced by stresses such as burn trauma are associated with a hypermetabolic/inflammatory state, that includes: increased protein catabolism (with resulting muscle wasting) and insulin resistance. Muscle wasting can lead to muscle weakness that can result in hypoventilation, prolongation of dependence on mechanical ventilation, prolonged rehabilitation and even death (16–19). Insulin resistance is a well established state in critically ill patients and plays a major role in metabolic derangements. Binding of insulin to its receptor (IR) activates the IR tyrosine kinase, which then phosphorylates IR substrates (IRSs). Phosphorylations of IRS1 and IRS2 transfer the signal from IR to phosphatidylinositol-3-kinase (PI3-kinase) (19,20). PTMs of the insulin signaling system are considered to be major disease-dependent events that regulate glucose transport via GLUT-4 translocation and downstream protein synthesis (21–27).

Akt1/PKBα is a critical downstream mediator of the IR/IRS/PI3-kinase pathway of the insulin signaling system (28–32). Akt1/PKBα consists of three structural features: the N-terminal pleckstrin homology (PH) domain, a large central kinase domain and a short C-terminal hydrophobic motif. High specific binding of the PH domain with membrane lipid products of PI3-kinase recruits Akt1/PKBα to the plasma membrane where phosphorylations of Thr^308^ (kinase domain) and Ser^473^ (hydrophobic motif) occur. Phosphorylation of Thr^308^ partially stimulates kinase activity; however, additional phosphorylation of Ser^473^ is required for full activity. Akt1/PKBα activation is associated with a disordered to ordered transition of a specific αC helix via an allosteric mechanism. A salt bridge between the side-chain of Lys^297^ and the phosphate group of pThr^308^ in this αC helix contributes to an ordered activation segment from DFG^292^ to APE^319^ (33–36). Reversible dephosphorylations of Thr^308^ and Ser^473^ by protein phosphatase 2A (PP2A) and PH domain leucine-rich repeat protein phosphatase (PHLPPα) also occur in the Akt1/PKBα activation/deactivation cycle (37–40).

In addition to the role of reversible phosphorylation/dephosphorylation in the regulation of Akt1/PKBα activity, this kinase is also reversibly inactivated by S-nitrosylation under conditions that result in a persistently increased production of NO (28,41–43); such as after burn injury as previously reported (44). Thiol titration and NMR data have indicated that a disulfide bond (Cys^60^-Cys^77^) exists in the kinase PH domain (45). A second disulfide bond in the kinase activation loop (Cys^297^-Cys^311^) has been reported to be associated with dephosphorylation under oxidative stress *in vitro* (46). In addition, it has been shown that when Cys^224^ of Akt1/PKBα is mutated to a Ser residue, the kinase becomes resistant to NO donor-induced S-nitrosylation and inactivation; suggesting that this residue is a major S-nitrosylation acceptor site (43). *In vivo* S-nitrosylations of the insulin receptor (β) and Akt1/PKBα result to reductions in their kinase activities (42). These data suggest that the redox status of Akt1/PKBα, regulated by NO, is a second factor in post-translational modifications that modulate kinase activity (via dynamic conformational changes) and thus GLUT-4 trafficking and protein synthesis.

Recent technical developments have made it feasible to study the molecular mechanisms of these important processes with quantitative proteomics. These techniques include: i) sensitive and site-specific procedures for the detection of S-nitrosylation based upon nano-LC interfaced with tandem MS/MS methods (47,48); ii) the Biotin-Switch method for thiol state discrimination between free, disulfide bonded and S-nitrosylated cysteine residues (49–54); problems associated with quantitative analysis by this technique have been discussed previously (48); iii) stable isotope labeling by amino acids in mammalians (SILAM) with partially ^15^N-labeled rats and completely L-^13^C_6_-Lys-labeled mice, and iv) highly specific anti-Akt1/PKBα monoclonal antibodies that can be used to immunoprecipitate the protein and yield clear SDS-PAGE bands with Coomassie Brilliant Blue R-250 staining.

Burn injury impairs IRS1 mediated signaling and attenuated IR-IRS-PI3K-Akt/PKB activation has been the focus of research studies at our Institute (22,24,26,28,41,43,44,47,50,57). Significantly reduced phosphorylations of Ser^473^ and Thr^308^, as well as Akt/PKB kinase activity were observed under burn injury [55% total burn surface area (TBSA), Day 3] and insulin simulation (41). Cys^296^ is found to be capable of capturing free radicals, such as NO, produced under burn injury (50). However, quantification of Cys^296^ PTM in the kinase loop remains a challenging issue.

To address these considerations and explore the feasibility of the basic SILAC approach in animals under acute phase and partial isotope labeling conditions, a mouse model with full-thickness 40% total burn surface area (TBSA) burn was used to proteomically characterize liver Akt1/PKBα from the perspectives of both protein expression and biological activity. (Isopropyl-^2^H_7_)-L-Leu was selected for labeling based on 4 considerations: i) L-Leu is an essential amino acid with high abundance in proteins. ii) compared with other amino acids, (isopropyl-^2^H_7_)-L-Leu is relatively inexpensive; iii) none of the 7 deuterium atoms of (isopropyl-^2^H_7_)-L-Leu are attached at the α-carbon which may be exchangeable with hydrogen atoms; thus in contrast to studies with (^2^H_10_)-Leu, MS offset can be eliminated as a confounding variable; iv) the hydrophobicity of L-Leu reduces false negative discovery during C18 reversed phase trapping and desalting.

## Materials and methods

### Chemicals

Acetonitrile (ACN, LC-MS Chromasolv), formic acid (FA), glacial acetic acid, LC-MS grade water, dithiothreitol (DTT), (Glu^1^)-fibrinopeptide B, *N,N*-dimethylformamide (DMF), were obtained from Sigma Chemical Co. (St. Louis, MO). SDS-PAGE ready gels (12% Tris-HCl, #161–1102), Laemmli sample buffer (#161–0737) and Coomassie Brilliant Blue R-250 (#161–0436) were obtained from Bio-Rad. The trypsin profile IGD kit (#PP0100) was obtained from Sigma. Anti-Akt1/PKBα monoclonal antibody (cat #05–796, Lot #26860) and inactive Akt1/PKBα (cat #14–279) were purchased from Upstate (Charlottesville, VA). Immobilized monomeric avidin beads were a product of Pierce Chemical Co., Rockford, IL (cat #20228). Iodoacetyl-LC-biotin (cat #21333) were purchased from Pierce.

### Burn injury

Male CD-1 mice (Charles River, Wilmington, MA) weighing 25–30 g were used throughout this study. The Institutional Animal Care Committee approved the study protocol. The animal care facility is accredited by the Association for Assessment and Accreditation of Laboratory Animal Care. A standardized thermal injury consisting of full-thickness scald involving 40% of the body surface area was performed on the mice (n=3) using previously described methods (55). Briefly, after anesthesia with intraperitoneal ketamine (60 mg/kg) and xylazine (1.3 mg/kg), the animals were shaven, and placed in a mold on top of a boiling water bath for 10 sec, exposing the back to a 40% body surface area full-thickness burn injury. Each mouse was given an intraperitoneal injection of saline (50 ml/kg) to replace intravascular volume known to be lost after thermal injury. Sham-treated (n=3, control) mice were anesthetized, confined to the mold without burning, dipped in water at room temperature, given saline resuscitation, and otherwise treated in the same fashion as the injured animals.

The routine protocol for pain management was 72 h of scheduled analgesia followed by p.r.n. analgesia based on overt signs of distress. This procedure was viewed as adequate pain management for this potentially painful procedure. The analgesic was buprenorphine (0.05 mg/kg s.c.) and/or a topical agent (EMLA cream) until discomfort disappeared. The buprenorphine was given 10–15 min prior to burn.

### Diets

The diet with depletion of L-Leu was obtained as L-amino acids defined diet for rats and mice. (Dyets, Inc., Bethlehem, PA; Dyet #510133, meets the 1995 NRC nutrient requirements). For producing the light diet, natural L-Leu was added to the diet powder (1% by weight) and producing the heavy diet, the same amount of (isopropyl-^2^H_7_)-L-Leu was added to the diet powder. For both diets, a minimal amount of water was added to make biscuits of adequate size. The biscuits were dried at room temperature for 1 week as suggested by Dyets in order to maintain the original nutrients.

### Metabolic labeling with (isopropyl-^2^H_7_)-L-Leu and natural L-Leu diets

Burned and sham-treated mice were caged separately and maintained in a temperature controlled facility with a 12-h light/dark cycle. Burned mice were fed with the diet containing (isopropyl-^2^H_7_)-L-Leu and sham-treated mice fed with the diet containing natural L-Leu. Five grams of light and heavy diets were provided daily to each mouse for 7 days. The animals had *ad libitum* access to water.

### Blood sampling

The mice were anesthetized with ether inhalation, and blood was drawn from the retro-orbital plexus. The blood samples were kept on ice and after centrifugation, serum samples were frozen in liquid nitrogen and stored at −80°C.

### Tissue homogenization

Equal amounts of tissue from burned and sham-treated animals were mixed and homogenized in cell lysis buffer (Cell Signaling Technology, cat #9803, 5 ml/g tissue) containing phenylmethanesulfonyl fluoride (BioChemika, 2 mM, freshly prepared 100X stock in ethanol just prior to use) on ice with 30 strokes. The homogenates were sonicated for 2 min at 4°C, followed by 3 freeze-thaw cycles in liquid nitrogen and 4°C. The homogenates (5 ml) were extracted with chloroform (1 ml) with vortexing for 15 sec and centrifuged at 14,000 x g for 2 min. Supernatants were collected and re-centrifuged at 14,000 x g for 2 min in order to remove traces of chloroform. The final supernatants were diluted with PBS to a final volume of 7 ml, and then passed through a membrane filter (0.22 μm, Millipore, cat # SLFG025NS). All tissue processing was performed at 4°C.

### Immunoprecipitation

Protein G bead slurry (50%, 100 μl) was transferred into an Eppendorf vial (1.5 ml), and washed 2 times with PBS at 4°C by gently rotating for 1 min. The packed beads (50 μl) were collated by centrifuging at 3000 x g (5000 rpm) for 20 sec. PBS (50 μl) was added to the beads to produce a final immunoprecipitation volume of 100 μl. Anti-Akt1 monoclonal antibody (5 μg, Upstate cat #05–796, clone AW24) was added and the mixture was gently stirred at 4°C for 1 h. Without washing the beads, processed homogenate was added and immunoprecipitation was performed for 2 h with gently rotating at 4°C.

### Biotinylation

Laemmli sample buffer (50 μl, 2X, pH adjusted to 8.0, Bio-Rad, cat #161–0737) was added to the washed immunocomplex beads (packed 50 μl, washed with 1 ml PBS 3 times for 5 min/cycle). This was followed by addition of freshly prepared iodoacetyl-LC-biotin solution (10 μl, stock solution; 2 mg in 1 ml of DMF). Cysteine acylation was performed at room temperature for 15 min with stirring. The reaction was quenched by addition of 2-mercaptoethanol (5 μl). The beads were then heated at 95°C for 5 min and kept at room temperature for 30 min prior to loading on SDS-PAGE gels.

### SDS-PAGE isolation and in-gel digestion

SDS sample solution (15 μl) was added to ready gel wells (Bio-Rad, 12% gel, cat #161–1102). SDS-PAGE was performed with Tris/Glycine/SDS running buffer (Bio-Rad, cat #161–0732) at 200 V for 45 min. The gels were removed and washed with deionized water for 5 min on a rocking platform and excess water was discarded. Proteins were stained with Coomassie Brilliant Blue R-250 for at least 1 h with gently shaking. The gels were washed three times with destaining solution (acetic acid:methanol:dH_2_O = 10:40:50) for 2 h. Protein bands were excised as 1x1 mm pieces, and placed into siliconized polypropylene Eppendorf tube (1.5 ml). In-gel digestion was performed with a Sigma Trypsin Profile IGD kit (cat #P0100) according to the manufacturer’s protocol.

### Avidin purification

Immobilized monomeric avidin beads (30 μl, 50% aqueous slurry, Pierce, cat #20228) were placed in siliconized polypropylene Eppendorf tubes (0.6 ml), and washed with PBS. The digested peptides (70 μl) were added to the packed avidin beads (15 μl) and the mixture was placed on a rocking platform for 30 min to capture the biotinylated peptides. The supernatant was collected, dried via Speed-Vac, and resuspended in mobile phase A (as described below, 15 μl) for control peptide ^252^FYGAEIVSALDYLHSEK^268^ analysis. The beads were then washed with PBS (200 μl x 3), followed by ACN/water (10/90 = v/v, 200 μl x 3). The biotinylated peptides were recovered by addition of formic acid (30 μl, 70%) and gently rocking for 5 min at room temperature. This recovery step was repeated three time and the supernatants were combined, dried via Speed-Vac, and resuspended in mobile phase A (15 μl) for biotinylated loop peptide ^290^ITDFGLCK^297^ analysis.

### LC-MS/MS analysis

All experiments were performed using a Waters CapLC-Q-TOF^micro^ system (Waters Corporation, Milford, MA). An analytical column (75 mm ID x 150 mm, C18 PepMap300, 5 mm, LC Packings) was used to connect the stream select module of the CapLC with the voltage supply adapter for ESI. Peptide mixtures were loaded onto the precolumn (C18 resin) at a flow rate of 15 μl/min. Dead volume from the CapLC injector to the precolumn was measured to be ~1.5 μl. After washing with mobile phase C (auxiliary pump, 0.1% formic acid in water/ACN, 2% ACN) for 2 min, the trapped peptides were back washed from the precolumn onto the analytical column using the 10-position stream switching valve. Freshly prepared mobile phases A and C were sonicated under vacuum for about 25 min and mobile phase B was treated in this way for 5 min. The mobile phases were degassed every week, and the CapLC pumps were wet primed for 20 cycles. A linear gradient was used to elute the peptide mixture from mobile phase A (0.1% FA in water/ACN, 2% ACN) to mobile phase B (0.1% FA in ACN). The linear gradient was segmented as follows: isocratic elution with 2% B for 3 min, increasing B from 10% to 70% (3–40 min) and isocratic 70% B for 5 min. Mobile phase B was then reduced to 2% over 2 min. The injector syringe (25 μl) was washed with degassed mobile phase A and the injection volume was set as full loop mode (10 μl). The gradient flow rate was set at 1.5 μl/min before the 16/1 Nanotee splitter and the pressure drop from the analytical column was about 800 psi. The pressure drop (or the flow splitting ratio) was adjusted and maintained with 20 μm ID capillary tubing at the waste outlet position of the Nanotee splitter. The gradient flow rate was ~95 nl/min. The electrospray voltage was set to ~3000 V to obtain an even ESI plume at the beginning of the gradient (high water content). As a routine sensitivity check, the PicoTip Emitter position and other parameters were adjusted to achieve ~45 counts/sec for the capillary tubing background peak (m/z 429). Sample cone and extraction cone voltages were set at 45 and 3 V, respectively. The instrument was operated in a positive ion mode with the electrospray source maintained at 90°C. The instrument was calibrated with synthetic human (Glu^1^)-fibrinopeptide B (100 fmol/μl in ACN/water = 10:90, 0.1% formic acid, v/v) at an infusion rate of 1 μl/min in TOF MS/MS mode. The peptide was selected at m/z = 785.8 and focused into the collision cell containing argon gas at ~3x10^−5^ Torr; the collision energy was set at 35 V. Instrument resolution for the (Glu^1^)-fibrinopeptide B parent ion, m/z = 785.84, was found to be 5,250 FWHM. All data were acquired and processed using MassLynx 4.1 software. Data-dependent acquisition (DDA) was set from m/z 630 to 660 for the biotinylated (doubly charged m/z 639.83) and control peptides (triply charged m/z 647.99). Scan time was ~1.9 sec and the inter-scan delay was 0.1 sec. MS to MS/MS switch criteria were dependent on reporter ion intensity (3 counts/sec) and detection window (2.3 Da, charge status). The instrument was switched from MS/MS back to MS after 5 sec without intensity restriction.

### Evaluation of the biotinylated Cys^296^ site

In contract to the highly abundant proteins in serum, biotinylated Cys^296^ is expressed at very low levels after burn injury and site-specific pinpointing requires careful evaluation of low S/N spectra. Confirmations of the biotinylated Cys^296^ residue were performed by the following three step procedure: i) for parent ion discoveries a narrow MS survey scan was used in order to increase MS sensitivity. Under these conditions, only a few false positive ions were observed and these were eliminated manually. ii) The positively discovered parent ions were analyzed with PepSeq of MassLynx V4.1 software. iii) For the loop peptide, with MS/MS scores <35 and S/N ~3, manual interpretations of candidate parent ions were performed with the following procedure: continuum MS/MS spectra were obtained and the upper 80% was centroided in order to obtain the sequence data associated with biotinylated y ions with expected mass shifts. Cysteine residue monoisotopic mass C_3_H_5_NOS = 103.01 Da was replaced with the iodoacetyl-LC-biotin derivatized adduct monoisotopic mass C_21_H_35_N_5_O_4_S_2_ = 485.21 Da.

## Results and Discussion

The essential issue for any isotope dilution method is to precisely characterize the heavy isotope-labeled internal standard in terms of atomic and chemical purities. To determine the relative quantification of liver Akt1/PKBα after burn injury, two typical serum acute phase proteins, negative regulated albumin and positive regulated amyloid P component, as well as liver Akt1/PKBα were characterized in a preliminary study with tissue and blood samples from 3 mice with burn injury (40% TBSA). To eliminate possible mathematical and biological complexities associated with of multiple isotopomer population distributions for individual tryptic peptides produced with partial labeling conditions, peptides with only one instance of (isopropyl-^2^H_7_)-L-Leu were selected for measurement of relative labeling efficiencies. A tryptic peptide may be positively charged at its N-terminal α-amine group and the side-chain amine group of a Lys residue or the guanido group of an Arg residue; thus, a doubly charged parent ion, (M+2H^+^), may be observed under ESI mass spectrometry. In addition, triply charged tryptic parent ions, (M+3H^+^), may be obtained for peptides containing proline or histidine residues or with larger size peptides. Peptides with one instance of (isopropyl-^2^H_7_)-L-Leu incorporation yield paired light and heavy parent ions with monoisotopic mass ion differences of 3.5 Da (doubly charged) or 2.3 Da (triply charged) under ESI conditions. Charge state dependent DDA allows these multiply charged peptides to be focused in the CID chamber under charged argon cleavages. The singly charged and predominated light and heavy MS/MS y ion series from the light and heavy parent ions are produced with mass difference of 7 Da. This allows unambiguous relative quantification of parent ion enrichments from possible false positive discoveries. The enrichments of three proteins are shown in Table I.

**Figure 1:**
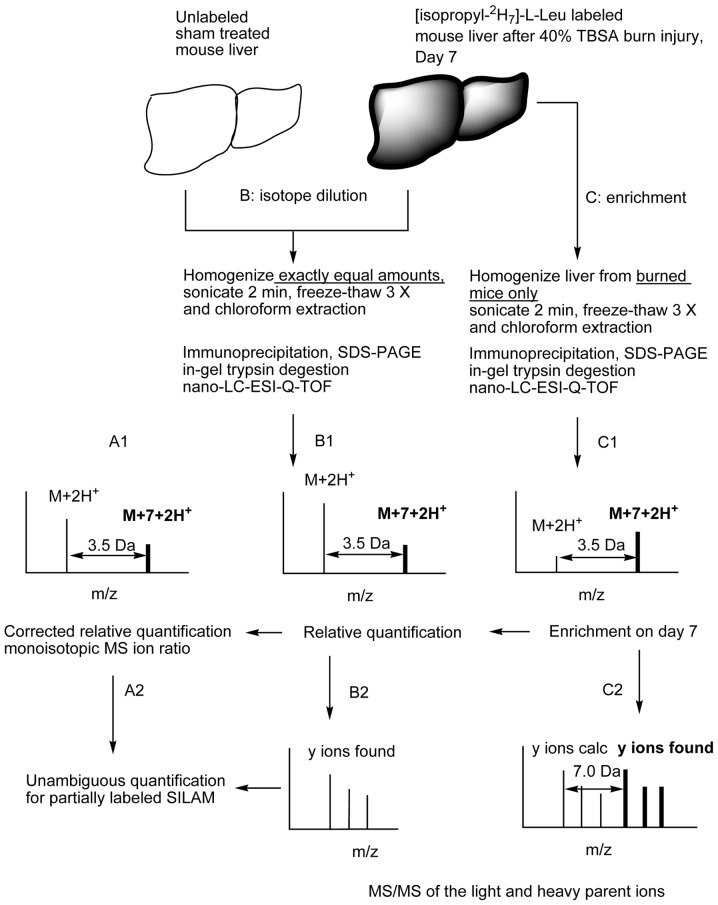
Schematic overview of the experimental workflow for partial metabolic labeling. Mice with full-thickness burn injury and sham-treated controls were maintained on diets containing (isopropyl-^2^H_7_) or natural L-Leu for 7 days. Liver tissue from the 2 groups of animals (n=3) were harvested and homogenized on Day 7. The homogenates were sonicated and subjected to 3 freeze-thaw cycles. Hydrophobic components were removed by chloroform extraction. A: corrected relative quantification for partially labeled SILAM. The intensity of unlabeled parent ion from burned animals (obtained by enrichment C1) was subtracted from the MS ion intensity of the corresponding light peptide after mixing the tissue samples. Unambiguous characterizations of partially metabolically labeled liver Akt1/PKBα were achieved with the isotope dilution method and MS/MS sequence information (A2, B2 and C2). B: relative quantification of liver Akt1/PKBα after burn injury. A mixture of the exact amounts (2 g each, isotope dilution) of liver from burned (heavy isotope labeled) and sham-treated (light isotope labeled) mice was homogenized and immunoprecipitated. The free cysteine residues were acetylated with iodoacetyl-LC-biotin at room temperature for 15 min followed by quenching with 2-mercaptoethanol. The beads were then heated at 95°C for 5 min and kept at room temperature for 30 min prior to SDS-PAGE. Biotinylated peptides, including the kinase loop peptide ^290^ITDFGLCK^297^, were captured with immobilized monomeric avidin beads. The control peptide, ^252^FYGAEIVSALDYLHSEK^268^ located just outside of the kinase loop (for assessment of kinase protein level after injury) was obtained from supernatant (B1). Unambiguous confirmations of the peptides and the expected labelings were obtained from singly charged y ions (B2). C: Akt1/PKBα enrichment determination. Akt1/PKBα was immunoprecipitated and free cysteine was biotinylated as described above. Doubly and triply charged tryptic peptides were analyzed with nano-LC interfaced with Q-TOF^micro^ tandem mass spectrometry (C1). Partially labeled parent ions with MS difference of 3.5 Da (doubly charged under ESI) were confirmed via their singly charged heavy y ions with MS difference of 7.0 Da (C2). The monoisotopic parent ion ratio of non-labeled (M+2H^+^) and labeled (M+7+2H^+^) peptides represents the metabolic labeling efficiency of one instance of (isopropyl-^2^H_7_)-L-Leu incorporation after burn injury. This is an updated proteomic version of classical isotope internal standard characterization.

The negative acute phase protein albumin and the positive acute phase protein amyloid P component had enrichments of ~55 and ~66%, respectively (Table I). The enrichment level (56%) of liver Akt1/PKBα was found to be very similar to that of albumin. These enrichment values represent ultimate (isopropyl-^2^H_7_)-L-Leu incorporation in each individual protein which can be used as the isotopic correction factor for the light and heavy parent ion MS ratio obtained by mixing exactly the same weight of labeled and non-labeled liver tissues (Fig. 1). The partially labeled SILAM reported here is a combination of classical isotope dilution principle and the updated nano-LC-ESI-Q-TOF approach.

**Figure 2:**
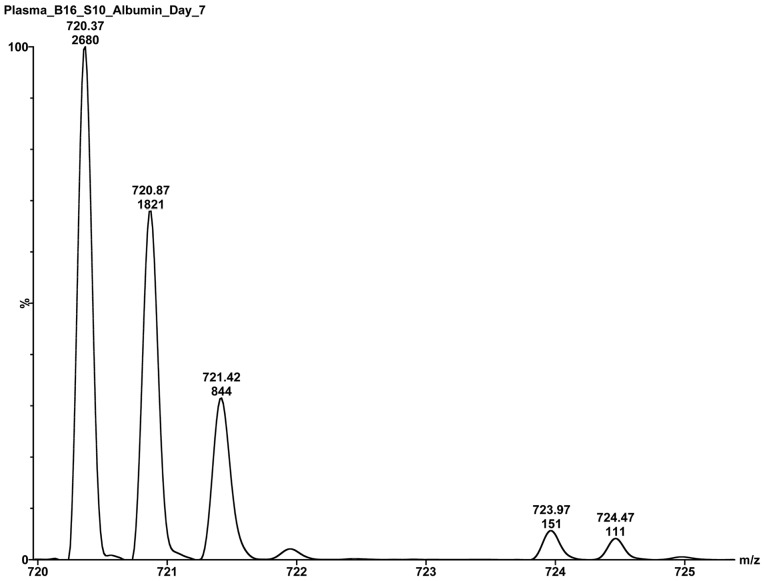
Relative quantification of the downregulation of serum albumin. The monoisotopic MS ion difference of 3.6 Da from the doubly charged parent ions at m/z 720.37 and 723.97 indicates (isopropyl-^2^H_7_)-L-Leu incorporation into the m/z 723.97 (^439^APQVSTPTLVEAAR^452^). MS/MS analysis confirmed that both ions have the same sequences. The corrected monoisotopic ion ratio, m/z 720.37: m/z 723.97 = 16.7 based on the enrichment listed in Table I, indicates that albumin was downregulated by 16.7 fold after burn injury.

**Figure 3:**
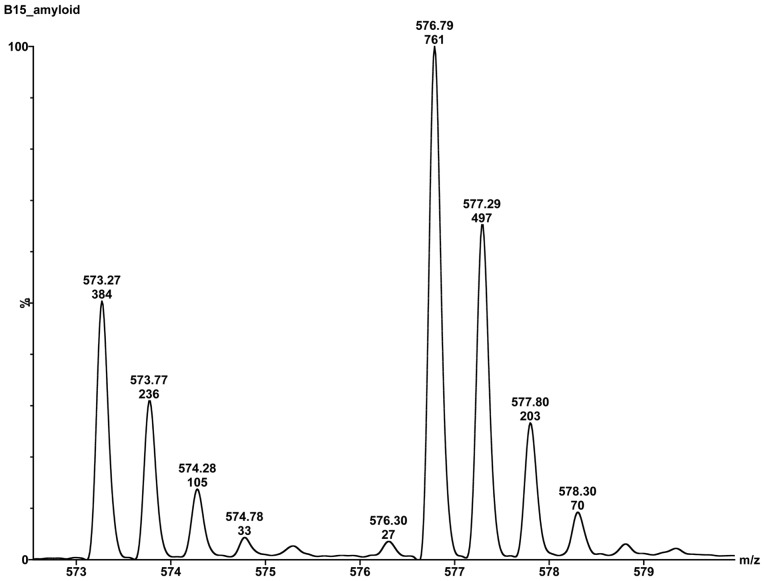
Relative quantification of the upregulation of amyloid P component. Amyloid P component light and heavy peptides (^66^SQSLFSYSVK^75^) were identified at m/z 573.27 (sham) and m/z 576.79 (burn) with doubly charged MS difference of 3.52 Da. A monoisotopic MS ion intensity ratio of m/z 573.27: m/z 576.79 = 0.5 demonstrated that there is little contribution of the m/z 573.27 from sham mice after mixing equal volumes of serums (2 μl). This suggests that amyloid P component is significantly upregulated (undetectable in sham-treated mice).

The ^439^APQVSTPTLVEAAR^452^ albumin light and heavy peptides, in response to the burn injury with one instance of the Leu residue for relative quantification are shown in Fig. 2. MS difference of 3.6 Da for the doubly charged monoisotopic MS ions at m/z 720.37 and 723.97 indicates (isopropyl-^2^H_7_)-L-Leu incorporation into the m/z 723.97 ion from metabolically labeled albumin after burn injury. MS/MS analysis confirmed that both parent ions have the same sequence and a 7.07 Da shift for the y6 ion (from m/z 658.39 to 665.46). The same m/z for the y5 ions (VEAAR) under sham (m/z 545.30) and burn conditions (m/z 545.31), indicates that, as expected, (isopropyl-^2^H_7_)-L-Leu incorporation labeled the y6 ion (LVEAAR). Also, the 54% enrichment of albumin (Table I), implies that approximately equal amounts of the ion at m/z 723.97 come from burned and sham animal sera. Thus, the ion intensity of 2680 under the m/z 720.37 peak needs to be subtracted (enrichment correction) by about the same ion intensity at m/z 723.97, i.e., the corrected m/z 720.37 is 2529. The corrected monoisotopic ion ratio, m/z 720.37: m/z 723.97 = 16.7, indicates that albumin was downregulated by 16.7 fold in the burned animals. In contrast, positively regulated amyloid P component reached a higher enrichment level of ~66%. Amyloid P component light and heavy peptides (^66^SQSLFSYSVK^75^) were found at m/z 573.37 (sham) and m/z 576.79 (burn) with doubly charged MS difference of 3.52 Da (Fig. 3). MS/MS data of the parent ion at m/z 576.79 indicates that the y6 ion (FSYSVK) without labeling occurs at m/z 695.47. However, heavy y7 ion (LFSYSVK) at m/z 815.61 was obtained with a mass increase of 7.02 Da compared with the corresponding unlabeled ion at m/z 808.59. The monoisotopic MS ion intensity ratio of m/z 573.27: m/z 576.79 = 0.5 indicated that there was little contribution of the m/z 573.27 ion from sham-treated mice after mixing equal volumes of serums (2 μl). In fact, Coomassie Brilliant Blue R-250 staining after SDS-PAGE did not demonstrate an amyloid P component band from serum (2 μl) from sham-treated animals. In this case, relative quantification failed due to lack of a control peptide; suggesting amyloid P component is upregulated significantly after the burn injury.

**Figure 4:**
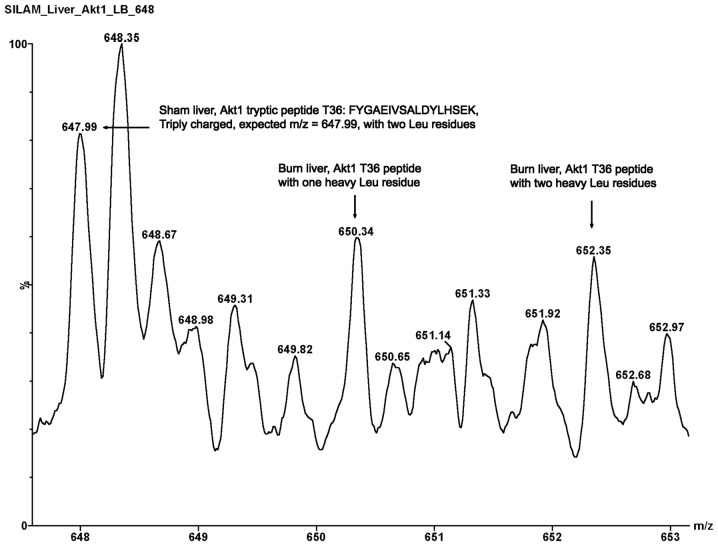
Changes in liver Akt1/PKBα expression after burn injury. A triply charged monoisotopic parent ion at m/z 647.99 was found for the control peptide ^252^FYGAEIVSALDYLHSEK^268^; calculated (M+3H^+^) = 647.99. Two heavy monoisotopic parent ions at m/z 650.34 and 652.36 indicate one and two instances of (isopropyl-^2^H_7_)-L-Leu incorporation into the control peptide. Akt1/PKBα protein expression, estimated from both heavy ions, was found to be upregulated after correction for both enrichment and background.

**Figure 5:**
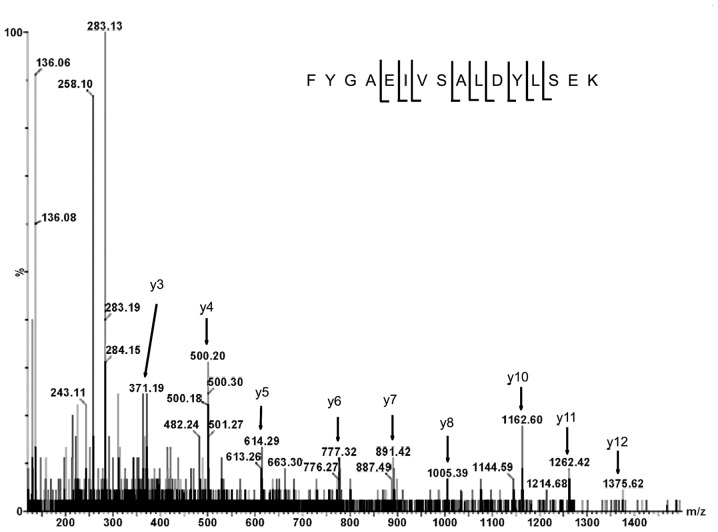
MS/MS sequencing of control peptides. MS/MS y ion series of the m/z 647.98 ion confirms the control peptide for studying protein expression.

**Figure 6:**
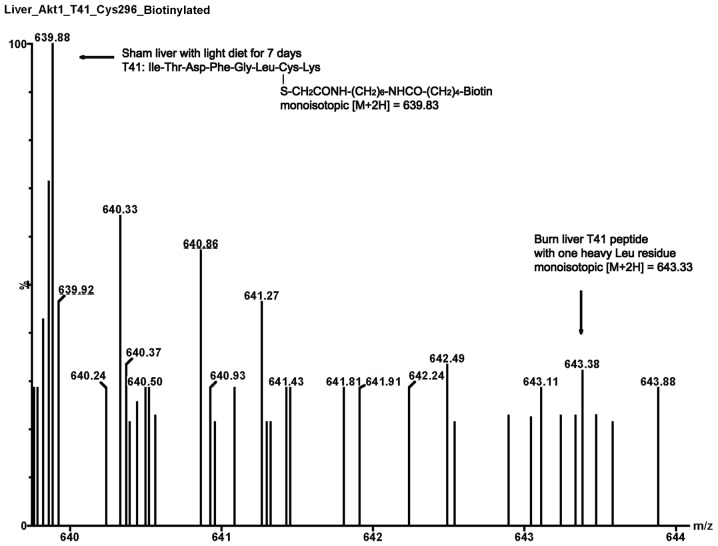
Liver Akt1/PKBα activity measurement via the loop peptide. Light peptide, ^290^ITDFGLCK^297^, of Akt1/PKBα from sham-treated mice were detected at m/z 639.88; doubly charged as indicated by the natural abundance carbon isotope peak at m/z 640.33. Biotinylated heavy peptides ^290^ITDFGLCK^297^ of Akt1/PKBα from the burn injured mice were detected at m/z 643.38; doubly charged as indicated by the natural abundance carbon isotope peak at m/z 643.88. The MS difference of 3.5 Da between the paired light and heavy parent ions indicates one instance of (isopropyl-^2^H_7_)-L-Leu incorporation into the heavy ion at m/z 643.38 after burn injury. The intensity of the m/z 643.38 ion indicates that the loop peptide containing Cys^296^ was markedly reduced (almost to baseline) in response to burn injury (n=3).

Akt1/PKBα was immunoprecipitated from the mixture of (isopropyl-^2^H_7_)-L-leucine-labeled liver from burned mice an equal amount tissue from sham-treated animals and processed further by the methods described above. The relative expression of Akt1/PKBα from livers of burned and sham-treated mice (Fig. 4). The triply charged monoisotopic parent ion at m/z 647.99 was detected for the control peptide ^252^FYGA EIVSALDYLHSEK^268^; calculated (M+3H^+^) = 647.99. Two heavy monoisotopic parent ions at m/z 650.34 and 652.36 indicates one and two instances of (isopropyl-^2^H_7_)-L-Leu incorporation into the control peptide. Triply charged MS differences were found to be 2.3 Da (calculated MS difference for one heavy Leu incorporation: 7/3=2.3 Da) and 4.3 Da (calculated MS difference for two heavy Leu incorporation: 14/3=4.6 Da), respectively. MS/MS y ion series (from y3 to y12 as shown in Fig. 5) of the m/z 647.98 confirms the control peptide for studying protein expression. The intensities of the m/z 650.34 and 652.36 ions represent the heavy Akt1/PKBα populations from burn injured mice, whereas the m/z 647.99 ion is derived from the sham-treated animals. Estimated total ion intensity was ~9.5 after burn injury and ~6.5 after sham treatment. Due to the low S/N of ~2 for the heavy ions and the enrichment of 56% in Table I, Akt1/PKBα protein expression, was observed to be upregulated in response to burn injury the mice. However, the heavy ion for the biotinylated peptide, ^290^ITDFGLCK^297^, which was used as a marker for kinase activity, was significantly reduced (almost to background) after injury (Fig. 6).

**Figure 7:**
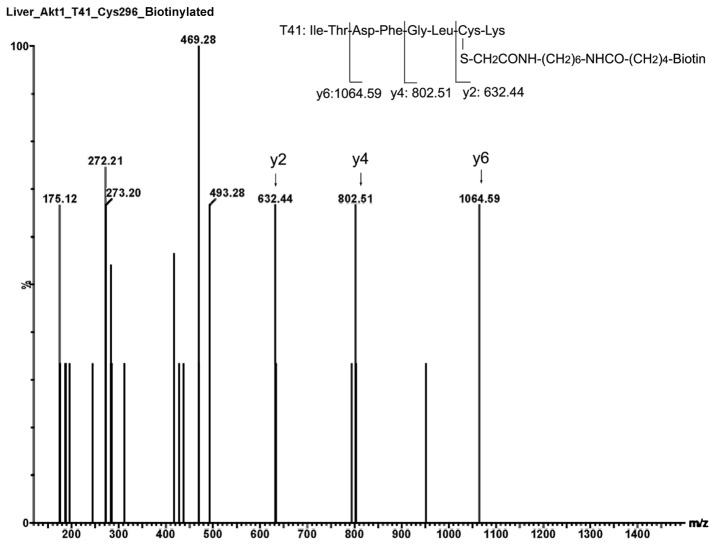
MS/MS sequencing of the kinase loop peptide. MS/MS spectra were smoothed and the upper 80% centroided to obtain sequence data in the cases of very low S/N. Three biotinylated y2, y4 and y6 ions at m/z 632.44, 802.51 and 1064.59 unambiguously confirmed the loop peptide sequence with biotinylated mass shifts for kinase bioactivity measurements.

The biotinylated light peptides ^290^ITDFGLCK^297^ of Akt1/PKBα from sham-treated mice was detected at m/z 639.88 and was doubly charged as indicated by the natural abundance carbon isotope peak at m/z 640.33. Three biotinylated y2, y4 and y6 ions at m/z 632.44, 802.51 and 1064.59 confirmed the loop peptide sequence with biotin modification (Fig. 7). The corresponding y2 ion of iodoacetyl-LC-biotin derivatized, ^296^CK^297^, was confirmed at m/z 632.44 (expected m/z 632.33 equal to 485.21 + 145.10 + 2.02). DDA with low S/N occurred *in vivo* studies was performed with a continuum mode to enhance the parent ion discovery. On the other hand, centroided spectra are found to be necessary to obtain accurately the diagnostic modifications in the y ions.

The biotinylated heavy peptide ^290^ITDFGLCK^297^ in Akt1/PKBα from burn injured mice (n=3) was identified at m/z 643.38 with its natural carbon isotopic peak at m/z 643.88. An MS difference of 3.5 Da between the paired light and heavy parent ions clearly indicated that one instance of (isopropyl-^2^H_7_)-L-Leu was incorporation into the doubly charged parent ion at m/z 643.38 with doubly charged state (n=3). MS ion intensity at m/z 643.38 indicated that loop peptide containing Cys^296^ was almost undetectable after burn injury. These observations were evaluated with MS/MS sequencing; peptide charge status and MS shift of the isotope-labeled paired peptides *in vivo*.

The incorporation efficiencies of isotopically labeled peptides in Table I demonstrates that amyloid P component was 10% higher than albumin; suggesting a faster turnover rate during upregulation. Serum albumin and liver Akt1/PKBα appear to have similar enrichments ~55%. Classically, individual protein fractional synthesis rate (FRS) is calculated by the relative isotope enrichment ratio of the labeled protein vs. precursor acyl-tRNA over the labeling time period for a given isotope tracer (56,57). FSR is a very important parameter for assessing effects of clinical interventions by comparisons between patients and healthy controls. In general, information about protein synthesis obtained with FRS and partially labeled SILAM are very similar and either can be used to optimize therapeutic strategies.

Our previous studies with thermally injured rats have demonstrated that there is no apparent alteration in binding of insulin to its receptors in liver, skeletal muscle or adipose tissue (22). Thus, acute and chronic insulin resistances induced by surgical trauma, burn injury, hemorrhage and sepsis are primarily post-receptor effects in the insulin-like growth factor/PI3-kinase/Akt pathway. Phosphorylations of specific Ser and Thr residues in the C-terminus of insulin receptor substrate-1 (IRS-1) induces its degradation via the ubiquitin-proteasome pathway (unpublished data); which may be an early biological effect after receptor binding. Impaired Akt1/PKBα kinase activity after injury may be a later downstream event. Deficiency of Akt1/PKBα causes decreased somatic cell and body size (58), while knockout of Akt2/PKBα leads to insulin resistance (59). Akt1/PKBα is involved in cellular survival pathways, by inhibiting apoptotic processes and stimulating protein synthesis pathways. It is also a key signaling protein in cellular pathways of skeletal muscle differentiation (60,61).

Currently, assays of Akt1/PKBα activity *in vitro* and *in vivo* are performed with antibodies specific for the phosphorylated Ser^473^ and Thr^308^ residues which are critical for stabilizing the global and loop active conformations of the kinase. Difficulties with using anti-phospho-Ser, but not anti-phospho-Tyr antibodies, have occurred in our phosphoproteomic research (unpublished data). NO production is elevated after burn injury and in patients with type 2 diabetic (61,63) and it has been shown that the Cys^297^-Cys^311^ disulfide bond in the kinase loop may be formed in association with dephosphorylation under oxidative stress *in vitro* (46). Reversible S-nitrosylation at Cys^296^ in the kinase loop is another PTM which complements reversible phosphorylation at Thr^308^ in the regulation of kinase activity (37). It has been shown that Akt1/PKBα undergoes transient phosphorylation/dephosphorylation which regulates the kinase active conformation cycle (37); kinase disulfide bond formation, Cys^297^-Cys^311^, and dephosphorylation at pThr^308^ are induced simultaneously by H_2_O_2_ oxidative stress *in vitro* (46); and high levels of nitric oxide production occurs in both burn injured rats (62) and diabetic patients (63). The free thiol group of Cys^296^ undergoes loop conformational changes by capture of nitric oxide, or chemical modifications with other reactive oxygen species produced under the burn injury; thus blocking substrate recognition. Intact loop peptide with a trace amount of free cysteine in the peptide population, ^290^ITDFGLCK^297^, after burn injury was developed as an unambiguous index for bioactivity. However, this peptide is not related to kinase protein expression in responses to the burn, since varying degrees of different thiol modifications in the loop may occur at the same time. In contrast, the control peptide, ^252^FYGAEIVSALDYLHSEK^268^, located just outside of the kinase loop, was a useful index of protein level.

Kinase bioactivity measured with the tandem MS was comparable with previously reported data measured with immune complex kinase assay and anti-pThr^308^ as well as anti-Ser^437^ monoclonal antibodies (41), whereas protein levels were slightly increased in responses to injury. MS/MS sequence analysis and (isopropyl-^2^H_7_)-L-Leu incorporation in the paired peptides indicated that after thermal injury kinase activity is significantly reduced, despite increased protein expression.

Overall, our findings indicate that neither complete labeling of nor measurement of acyl-tRNA enrichment are necessary or critical for quantitative proteomics with SILAM. Cys^296^ thiol state is considered as one of the important factors for the kinase activity. The limitations of partially labeled SILAM for clinical studies are first that specific protein enrichment must be measured in tissues labeled with heavy isotopes under stress conditions; unfortunately, many proteins of clinical interest occur at low abundance and thus there is a high rate of false negative discovery. Second, sequence confirmation of individual proteins requires that SDS-PAGE bands be visible by Coomassie Brilliant Blue R-250 staining (at least 0.1 μg). Despite these limitations our observations may provide new insights into the treatment of muscle wasting and other aspects of insulin resistance after critical injury.

## Figures and Tables

**Table I:** Protein enrichment levels on Day 7 after third degree burn of 40% total burn surface area.

	Sequence, (charge state observed)	Enrichments %, (SD)
Albumin	^243^LSQTFPNADFAEITK^257^, (2)	53 (3.2)
	^559^HKPKATAEQLK^669^, (2)	57 (3.8)
	^439^APQVSTPTLVEAAR^452^, (2)	54 (4.1)
Amyloid P component	^66^SQSLFSYSVK^75^, (2)	66 (4.9)
	^88^VGEYSLYIGQSK^99^, (2)	65 (4.6)
	^147^APPSIVLGQEQDNYGGGFQR^166^, (3)	69 (5.2)
Liver Akt1/PKBα	^9^EGWLHKR^15^, (2)	59 (4.0)
	^184^EVIVAKDEVAHTLTENR^200^, (3)	56 (2.7)
	^290^ITDFGLB^*^KEGIKDGATMK^307^, (3)	55 (3.3)

B^*^, 2-mercaptoethanol derivatized cysteine; L, partially labeled L-Leu residues.

## Abbreviations

Akt1/PKBα: Akt1/protein kinase Bα
CID: collision induced dissociation
DDA: data-dependent acquisition
FSR: fractional synthesis rate
MS/MS: mass spectrometry/mass spectrometry
PTM: post-translational modification
Q-TOF tandem MS: quadruple time-of-flight tandem mass spectrometry
SILAM: stable isotope labeling by amino acids in mammalians
TBSA: total burn surface area
